# Improved sensitivity of the urine CAA lateral-flow assay for diagnosing active *Schistosoma* infections by using larger sample volumes

**DOI:** 10.1186/s13071-015-0857-7

**Published:** 2015-04-22

**Authors:** Paul LAM Corstjens, Ruth K Nyakundi, Claudia J de Dood, Thomas M Kariuki, Elizabeth A Ochola, Diana MS Karanja, Pauline NM Mwinzi, Govert J van Dam

**Affiliations:** Department of Molecular Cell Biology, Leiden University Medical Center, Einthovenweg 20, P.O. Box 9600, 2300 RC Leiden, the Netherlands; Institute of Primate Research, National Museums of Kenya, Nairobi, Kenya; Center for Global Health Research, Kenya Medical Research Institute, Kisumu, Kenya; Department of Parasitology, Leiden University Medical Center, Leiden, the Netherlands

**Keywords:** Schistosomiasis, Circulating anodic antigen (CAA), Active infection, Transmission interruption, Elimination, Diagnostic test, Lateral flow (LF), Upconverting phosphor (UCP)

## Abstract

**Background:**

Accurate determination of *Schistosoma* infection rates in low endemic regions to examine progress towards interruption of transmission and elimination requires highly sensitive diagnostic tools. An existing lateral flow (LF) based test demonstrating ongoing infections through detection of worm circulating anodic antigen (CAA), was improved for sensitivity through implementation of a protocol allowing increased sample input. Urine is the preferred sample as collection is non-invasive and sample volume is generally not a restriction.

**Methods:**

Centrifugal filtration devices provided a method to concentrate supernatant of urine samples extracted with trichloroacetic acid (TCA). For field trials a practical sample volume of 2 mL urine allowed detection of CAA down to 0.3 pg/mL. The method was evaluated on a set of urine samples (n = 113) from an *S. mansoni* endemic region (Kisumu, Kenya) and compared to stool microscopy (Kato Katz, KK). In this analysis true positivity was defined as a sample with either a positive KK or UCAA test.

**Results:**

Implementation of the concentration method increased clinical sensitivity (Sn) from 44 to 98% when moving from the standard 10 μL (UCAA10 assay) to 2000 μL (UCAA2000 assay) urine sample input. Sn for KK varied between 23 and 35% for a duplicate KK (single stool, two slides) to 52% for a six-fold KK (three consecutive day stools, two slides). The UCAA2000 assay indicated 47 positive samples with CAA concentration above 0.3 pg/mL. The six-fold KK detected 25 egg positives; 1 sample with 2 eggs detected in the 6-fold KK was not identified with the UCAA2000 assay.

**Conclusions:**

Larger sample input increased Sn of the UCAA assay to a level indicating ‘true’ infection. Only a single 2 mL urine sample is needed, but analysing larger sample volumes could still increase test accuracy. The UCAA2000 test is an appropriate candidate for accurate identification of all infected individuals in low-endemic regions. Assay materials do not require refrigeration and collected urine samples may be stored and transported to central test laboratories without the need to be frozen.

## Background

Interruption of transmission of *Schistosoma* infections and potential elimination is thought feasible as highlighted by the commitment of the 65th World Health Assembly [1]. Several approaches are being evaluated to develop methods to move forward, including mass drug administration programs, improved sanitation and availability of clean water, as well as snail control [2-6]. To monitor the effect of these efforts, and to identify remaining pockets of infection, highly accurate diagnostics are obligatory especially for identification of individuals with very low levels of infection [7-10]. In most populations, serology (antibody detection) is not the appropriate method to detect active infections as it will also indicate past infections [11,12]. Detection of the presence of living worms can be done by showing schistosome eggs, nucleic acid, or antigens in different human samples. Importantly, one needs to consider the time metabolic and biological mechanisms require to clear these components from the human body after drug treatment. Excretion of schistosome eggs continues for weeks [13] and therefore does not allow a diagnosis of the efficiency of treatment shortly after drug administration; much more rapidly cleared circulating schistosome-antigens in that respect present a better potential [14-16].

Since many years, the still widely applied method to diagnose active infection in low resource settings is detection of eggs in urine (*S. haematobium*) or stool (*S. mansoni* and *S. japonicum*), although this method is elaborate and lacks sensitivity [17]. For intestinal *S. mansoni* infections, a point-of-contact rapid urine-based antigen test (POC-CCA; Rapid Medical Diagnostics, Pretoria, RSA) is available which is based on the detection of a worm regurgitated antigen (circulating cathodic antigen, CCA). The sensitivity of the POC-CCA test has been reported to be better than or equivalent to stool egg counts based on a triplicate Kato-Katz analysis [18]. In case of *S. haematobium*, causing urogenital schistosomiasis, potential infections are indicated by microhematuria leading to small amounts of blood in the urine detectable with a rapid dipstick test [19]. Note that specificity of latter test for schistosomiasis is inadequate as it cannot rule out other urinary-tract infections. More importantly, urine POC-CCA and microhematuria dipstick are not sensitive enough to determine true prevalence of infection in near-elimination settings where transmission appears to be interrupted. Also, prevalence based on egg counts is expected to significantly underestimate true infection rates as illustrated by the statistical model of de Vlas *et al.* [20]. A highly sensitive and increasingly applied method to detect pathogen infections is by means of PCR, detecting nucleic acids. This has been demonstrated using urine and stool samples for the major human *Schistosoma* infections [21-25] and has excellent multiplex capability (simultaneous screening for and detection of multiple infections/pathogens). However, this technology is very expensive and not readily available for resource limited setting and remote areas. Moreover, nucleic acid based assays mostly detect egg DNA and similar to egg counts have some limitation in testing effects of drugs shortly after administration.

Earlier described antigen assays have demonstrated to be well suited for detection of active infections (see refs in Corstjens *et al.* [26]). Initial ELISA formats have been converted to user-friendly lateral-flow based assays [27,28]. The POC-CCA urine strip test is the only commercially available lateral flow test applied for routine detection of *S. mansoni* infections. Although for low endemic settings the sensitivity is still too low, the test is well suitable as a mapping tool and appropriate for monitoring and evaluation in schistosomiasis control programmes [18] as an alternative for microscopic stool analysis. Another, more advanced antigen test detects the *Schistosoma* circulating anodic antigen (CAA). The in this study described CAA test is genus-specific allowing detection of all *Schistosoma* species including veterinarian (see references in Corstjens *et al.* [26]). As an assay, it has a somewhat higher complexity which mainly relates to a sample pretreatment step, an extraction with trichloroacetic acid (TCA) followed by a centrifugation step. The resulting clear supernatant containing the targeted CAA (a carbohydrate structure) is then analysed on a LF strip utilizing a unique fluorescent reporter, upconverting phosphor (UCP) particles [29]. A previously developed, robust, user-friendly dry-reagent format of the UCP-LF CAA assay [30] allowing convenient shipping and storage at ambient temperature, has already been utilised by local staff in different laboratories around the world [26,31-33]. In the current study we demonstrate further improvement of the sensitivity of the UCP-LF CAA assay by including a concentration step in the sample pretreatment protocol as proposed previously [26]. This step involves the concentration of the TCA-soluble fraction of urine samples using Amicon Ultra Centrifugal Filter Devices (Millipore Corp.), allowing larger sample input, increasing the volume from 10 μL urine to 250 and 2000 μL, or even 7500 μL. The results confirm that the larger sample input identified samples with CAA concentrations well below the 30 pg/mL cutoff threshold as maintained for the standard dry-reagent UCP-LF CAA assay without concentration and requiring only 10 μL urine (UCAA10) [26]. For the sample set tested in this study, when using the 2000 μL urine concentration assay (UCAA2000) the prevalence increased to the level that would be predicted based on eggs detected in stool samples following the model of de Vlas *et al.* [20].

## Methods

### Ethics statement

This study was reviewed and approved by the scientific steering committee and the national ethics review committee of the Kenya Medical Research Institute. Parents of minors provided written informed consent and minors provided written assent to participate in the study. All positive cases were treated appropriately.

### Study site

Samples were collected from a village near Kisumu, Western Kenya, along the shores of Lake Victoria where only intestinal schistosomiasis is endemic. There were no records or other indications for urogenital schistosomiasis. Previous screenings for microhaematuria always showed less than 2% positives for potential urinary tract infections.

### Collection of stool specimen and Kato-Katz analysis

Participants were provided with stool containers and asked to provide fresh stool samples on three consecutive days. Samples were transported to the KEMRI/CDC laboratory where they were processed and examined using the Kato-Katz technique [34] for detection of parasite eggs, two slides (42 mg) per stool. Internal quality control was achieved by having different microscopists reading both slides, with each technician blinded to the other corresponding stool result. The arithmetic mean of egg counts was calculated from the total slides per child, and expressed as eggs per gram (epg).

### Collection of urine specimens and storage

On day one of the stool collection, fresh morning mid-stream urine was collected from school children into clean pre-labelled tubes to ensure it was free from any contaminants. Hematuria, indicating potential *S. haematobium* infection, was determined using URiSCAN test strips (YD Diagnostics, Kyunggi-Do, Korea). Urine samples were split, one part stored at −20°C and the other part stored at ambient temperature for three weeks and transported to IPR (Nairobi) where they were tested with the dry-reagent UCP-LF CAA assays (UCAA10, UCAA250 and UCAA2000).

### UCP-LF CAA assays

For the standard UCAA10 assay, urine samples were extracted with an equal volume of 4% w/v TCA as described previously [28]. Of the resulting TCA-soluble fraction (TCA-supernatant, consisting of 50% v/v urine, 2% w/v TCA), 20 μL was added to a tube containing 100 ng of dry CAA-specific UCP reporter (400 nm Y_2_O_2_S:Yb^3+^,Er^3+^ particles [29] coated with mouse monoclonal anti-CAA antibodies) hydrated in 100 μL assay buffer (100 mM HEPES pH 7.5, 270 mM NaCl, 0.5% v/v Tween-20, 1% w/v BSA) [30]. Tubes were incubated for 60 min at ambient temperature (25–30°C) in an orbital shaker (900 rpm); note that the standard protocol indicated incubation at 37°C [30]. Next, immunochromatography was initiated by placing a CAA-specific LF strip [28] in the tube and LF allowed to continue until strips were dry. LF strips were analysed using a lightweight portable UCP scanner (UCP-Quant [35,36]; QIAGEN Lake Constance GmbH, Stockach Germany). Test line signals (T) were normalized to flow control signals (FC) of the individual LF strips and results expressed as a ratio value (T/FC) [37,38]. Serial dilutions, prepared in normal human urine (NHU), of TCA-soluble fraction of *Schistosoma* adult worm antigen (AWA-TCA; containing 3% w/w CAA [26]) were analysed in parallel to provide standard curves for calculation of the CAA concentrations in the clinical samples.

For the UCAA250, −2000 and −7500 concentration assays, 500, 4000 or 15000 μL TCA-supernatant was concentrated to 20 μL using 0.5, 4, and 15 mL centrifugal filter devices (Amicon Ultra-0.5, −4, −15 Centrifugal Filter Devices; Millipore Corp.), respectively. The concentrates were then processed as described for the UCAA10 assay.

The during production maintained quality control (QC) thresholds for the dry-reagent UCP-LF CAA assays were 30, 3, 0.3 and 0.1 pg CAA per mL urine for the UCAA10, −250, −2000 and −7500 assay, respectively [26]. An indecisive range was defined of 30–15, 3–1.5, 0.3-0.15 and 0.1-0.05 pg/mL, respectively (note that in-house at LUMC, UCP-LF CAA assays may be performed with wet UCP reagents which require a sonication step but have a 3-fold better analytical sensitivity [26]). Clinical samples generating test results with CAA concentrations falling in the indecisive range are potentially positive, but if interpreted as negative would result in 100% specificity. Samples with test results below the lower value of the indecisive range are identified as negatives. The UCAA7500 assay was not included in the testing of the clinical set of urine samples.

## Results and discussion

### Stability of the CAA in urine

**Figure 1:**
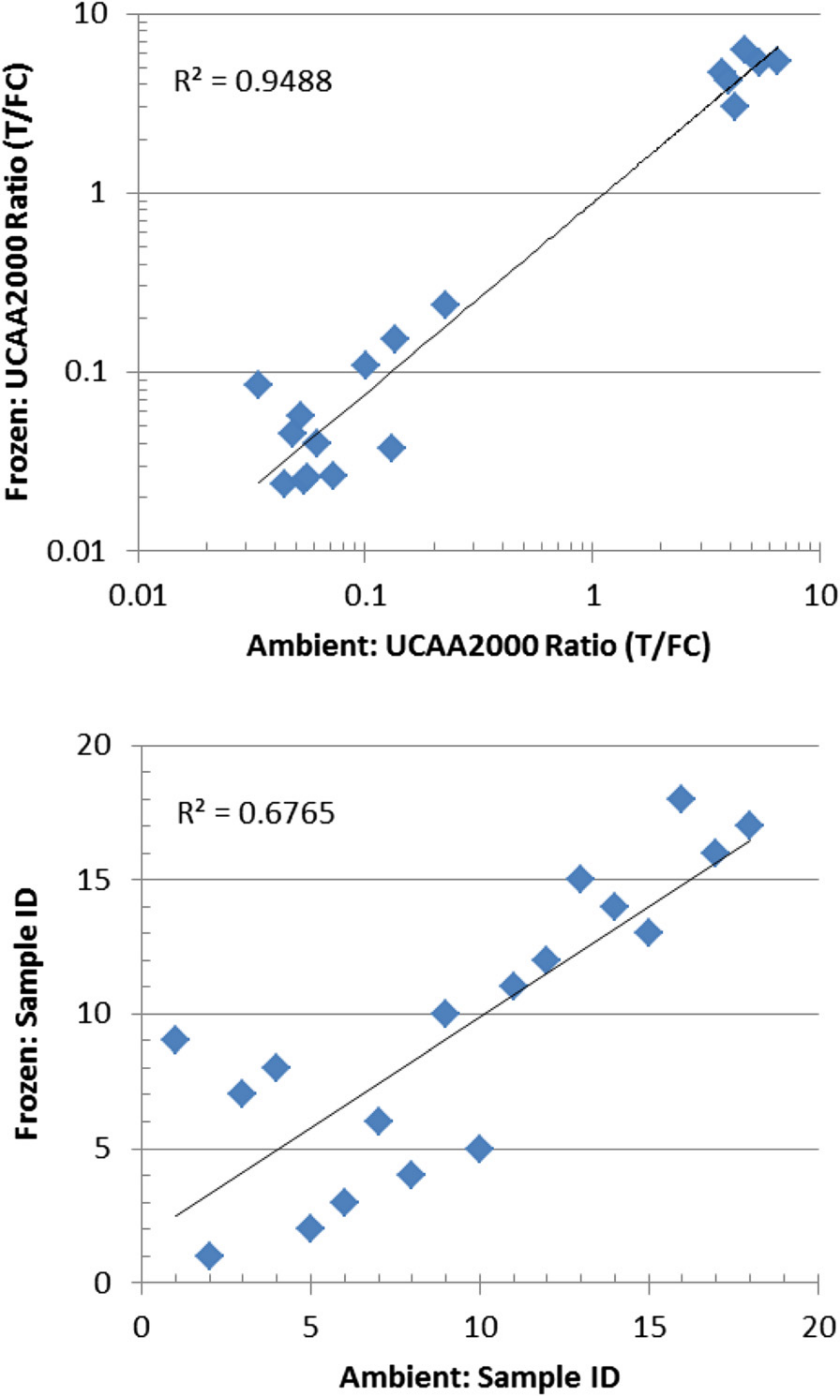
UCAA test results of corresponding urine samples stored frozen or at ambient temperature. Upper panel: scatter plot showing the Pearson correlation of the UCAA2000 assay results presented as ratio value (T/FC). Lower panel: Spearman ranking of the samples also based on T/FC ratio value.

Previous testing in our laboratory indicated that storage conditions of the urine samples did not require highly controlled conditions (unpublished). CAA concentrations were not different in matching urine samples that were stored either at ambient temperature for a few weeks, refrigerated at 4°C for several months, or frozen at −20°C for years. The current study included storage of a small set (n = 18) of urine samples at ambient temperature (25–35°C) for 3 weeks, compared to storage in the freezer (−20°C). This three weeks period was chosen as it would allow convenient collection of urine samples in field experiments with subsequent transport to central laboratories without the need for a cold chain. Prior to use, (thawed) urine samples were mixed with an equal volume of 4% (w/v) TCA. Precipitates and any other particulate material in this mixture are removed by centrifugation; note that this step also takes care of potential precipitates formed as a consequence of storage. Figure 1 shows the association of the CAA concentrations for the two storage conditions as a Pearson correlation (upper panel) and a Spearman ranking scattergram (lower panel). CAA levels are determined with the UCAA2000 assay and arbitrarily expressed by UCP-LF assay values (T/FC ratio). The good association of this small set indicates high stability of the CAA component in urine samples kept at ambient temperature for several weeks which may allow simplified sample storage and shipping conditions.

### Implementation of concentration devices – improved limit of detection

**Figure 2:**
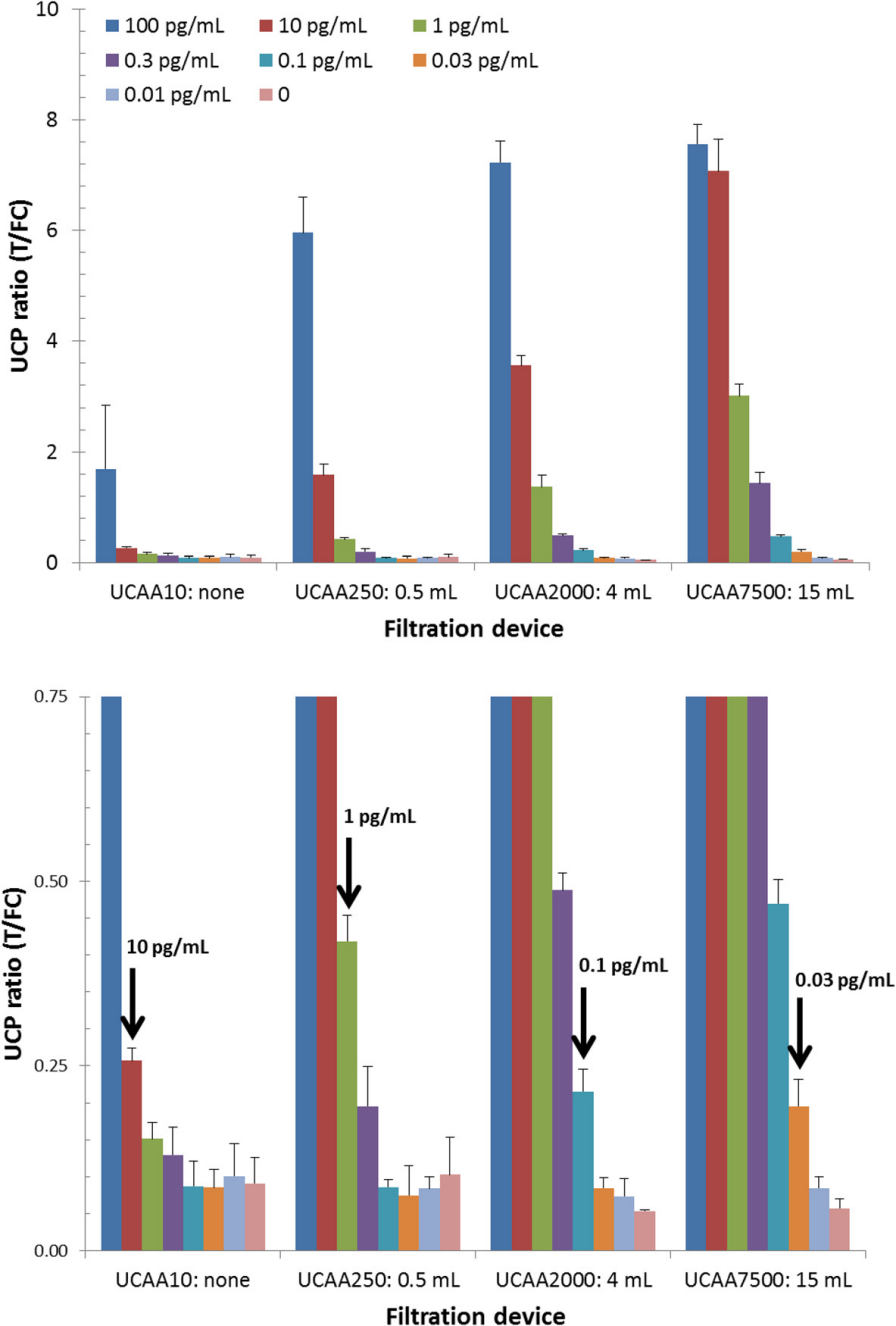
Effect of increased sample input on the LLOD of the UCP-LF CAA assay. AWA-TCA standard series in urine (CAA concentration indicated in upper panel), analysed in triplicate with the UCP-LF wet assay format. Spiked urine was extracted with 1 volume 4% (v/v) TCA, the resulting TCA-supernatant was either tested directly with the UCAA10 assay or first concentrated using 0.5, 4, and 15 mL Amicon centrifugal filter devices. Arrows in the lower panel (a blow up of the y-axis) indicate the QC cutoff threshold for the different assays; left to right: UCAA10, −250, −2000 and −7500. Error bars indicate 1 standard deviation (n = 3).

The standard UCP-LF CAA assay for urine (UCAA10) allows testing of 20 μL TCA-supernatant (50% v/v urine, 2% w/v TCA) with a pre-determined QC cutoff threshold of 10 or 30 pg CAA per mL, respectively for the wet- and dry-reagent format. To improve the lower limit of detection (LLOD), TCA-supernatant was concentrated using centrifugal filter devices. Assuming a single load protocol (meaning that the Amicon concentration devices were loaded ‘only’ once with the indicated maximum capacity), the assessed 0.5, 4 and 15 mL centrifugal devices theoretically would allow a 25-, 200- and 750-fold concentration, respectively. A standard series of AWA-TCA in NHU was analysed in triplicate with the UCAA10, −250, −2000 and −7500 using a freshly sonicated stock solution of UCP reporter particles (referred to as wet assay format [28]). For this format, the LLOD detection of CAA in urine progressed from 10 pg/mL to 0.03 pg/mL when increasing urine sample input from 10 μL to 7.5 mL; a practical improvement by 50% of the theoretical limit (Figure 2). Similar factors were achieved with the dry-reagent assay (UCP particles supplied as a dry pellet [30]). When using the UCAA7500 assay, 15 mL TCA-supernatant (containing 7.5 mL urine) is first concentrated to 500 μL with a 15 mL centrifugal filtration device and then transferred to a 0.5 mL centrifugal device for further concentration to 20 μL. The approximate centrifugation time required for the different devices is 15, 30 and 60 minutes for the 10 kDa molecular weight cutoff filtration devices used in this study.

### Analysis of the ***S. mansoni*** Kisumu urine set with UCP-LF CAA assay

**Figure 3:**
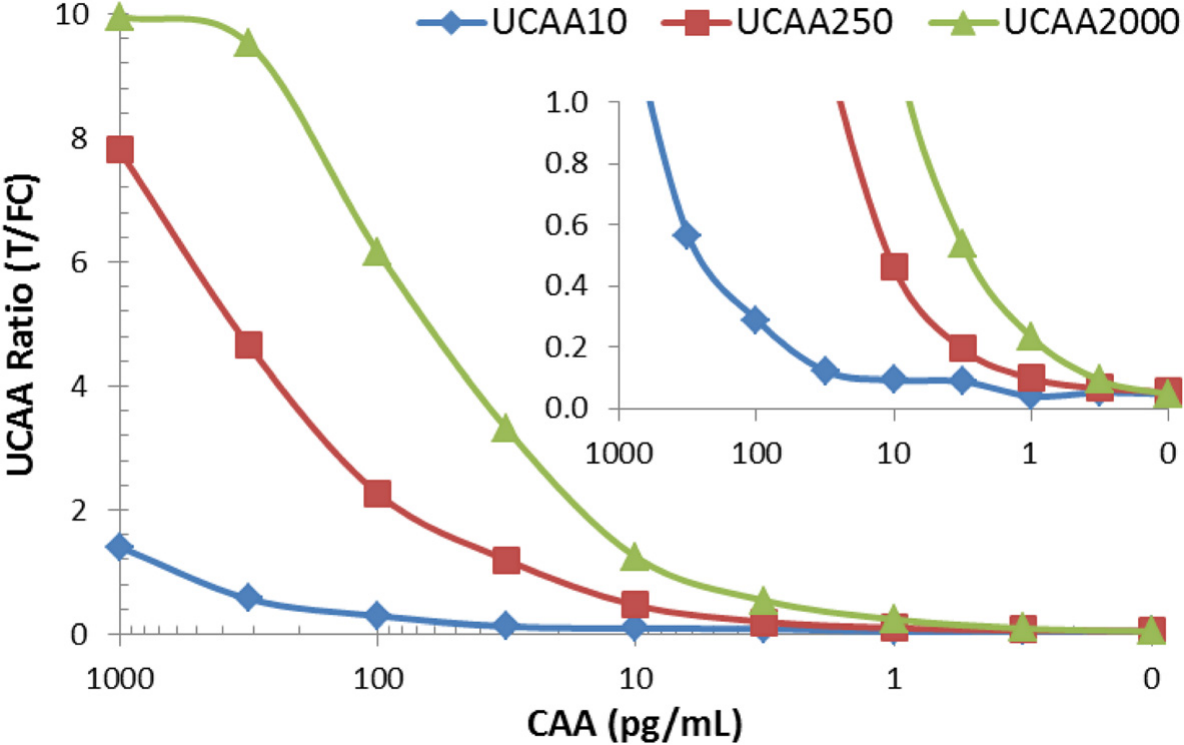
CAA standard series in urine analysed with the UCP-LF concentration assays. The amount of urine analysed per strip increased from 10, 250 to 2000 μL, respectively for the UCAA10, −250 and −2000 assay. The UCAA250 and UCA2000 require concentration of the TCA-supernatant using Amicon Centrifugal Filter Devices Ultra-0.5 and Ultra-4, respectively.

The urine samples collected in Kisumu were tested at IPR (Nairobi) with the UCP-LF dry-reagent assay. These assay materials were transported from the Netherlands to Kenya without cooling, and on arrival stored at ambient temperature. Not needing a cold chain for shipping was an important feature that reduced cost and logistic issues. All 113 samples were tested (in singlet) with the UCAA10 and −250 assay. However, material restrictions only allowed testing of 73 samples with the UCAA2000 assay. Scanning of the LF strips and analysis of the result was also performed at IPR utilizing a lightweight reader with appropriate software as described for other studies [35]. CAA concentrations were determined from standard curves obtained with NHU spiked with AWA-TCA tested with the UCAA10, −250 and −2000 assay (Figure 3) using a 4 parameter curve fit method. Samples were classified as positive, indecisive or negative according the predetermined QC cutoff thresholds. The number of CAA positives increased from 21 (identified with the UCAA10 assay) to 30 when using the concentration based assays UCAA250. In the subset of 73 samples that were tested with all 3 UCAA assays, the number of CAA positives increased from 15 in the UCAA10, to 20 in the UCAA250, to 31 in the UCAA2000, respectively. Increased sample volume obviously did result in an increase of the number of identified CAA positives, raising the percentage positives in the (non-random) subset from 19%, to 27%, to 42% for the respective assays (Table 1). Moreover, positives identified by the UCAA10 assay were all confirmed with the UCAA250 assay, and similarly from the UCAA250 assay to the UCAA2000 assay. Out of the 6 UCAA10 indecisive samples 50% was a true positive as demonstrated with the larger sample volume assay (UCAA250). The single sample classified indecisive with the UCAA250 did not test positive with the UCAA2000 assay. We speculate that the percentage of indecisive samples that actually turn out positive may vary per setting. The pre-determined QC cutoff thresholds firmly maintain 100% specificity. Depending on the objective of the study, when targeting 100% sensitivity the indecisive group should be included in the positive group.

**Table 1:** **Effect of increased sample input on the number of CAA positives**

***Assay result*** ^***a***^	***UCAA10***	***UCAA250***	***UCAA10***	***UCAA250***	***UCAA2000***
***Positive***	21 (19%)	30 (26%)	15 (19%)	20 (28%)	31 (43%)
***Indecisive***	6 (5%)	1 (1%)	6 (5%)	1 (1%)	3 (4%)
***Negative***	86 (76%)	82 (73%)	52 (71%)	52 (71%)	39 (53%)
***Samples tested*** ^***b***^	113	113	73	73	73

^a^Numbers represent the amount of samples with a specific test result (between brackets as the percentage of total number of samples tested).

^b^The Kisumu sample set comprised urine samples of 113 individuals which were tested with the UCAA10 and UCAA250 assay. A non-random subset of 73 samples was also tested with UCAA2000 assay (not included in this subset were 10 samples that scored a positive test result with both, UCAA10 and UCAA250, plus 30 samples that scored a negative test result with the UCAA250).

**Figure 4:**
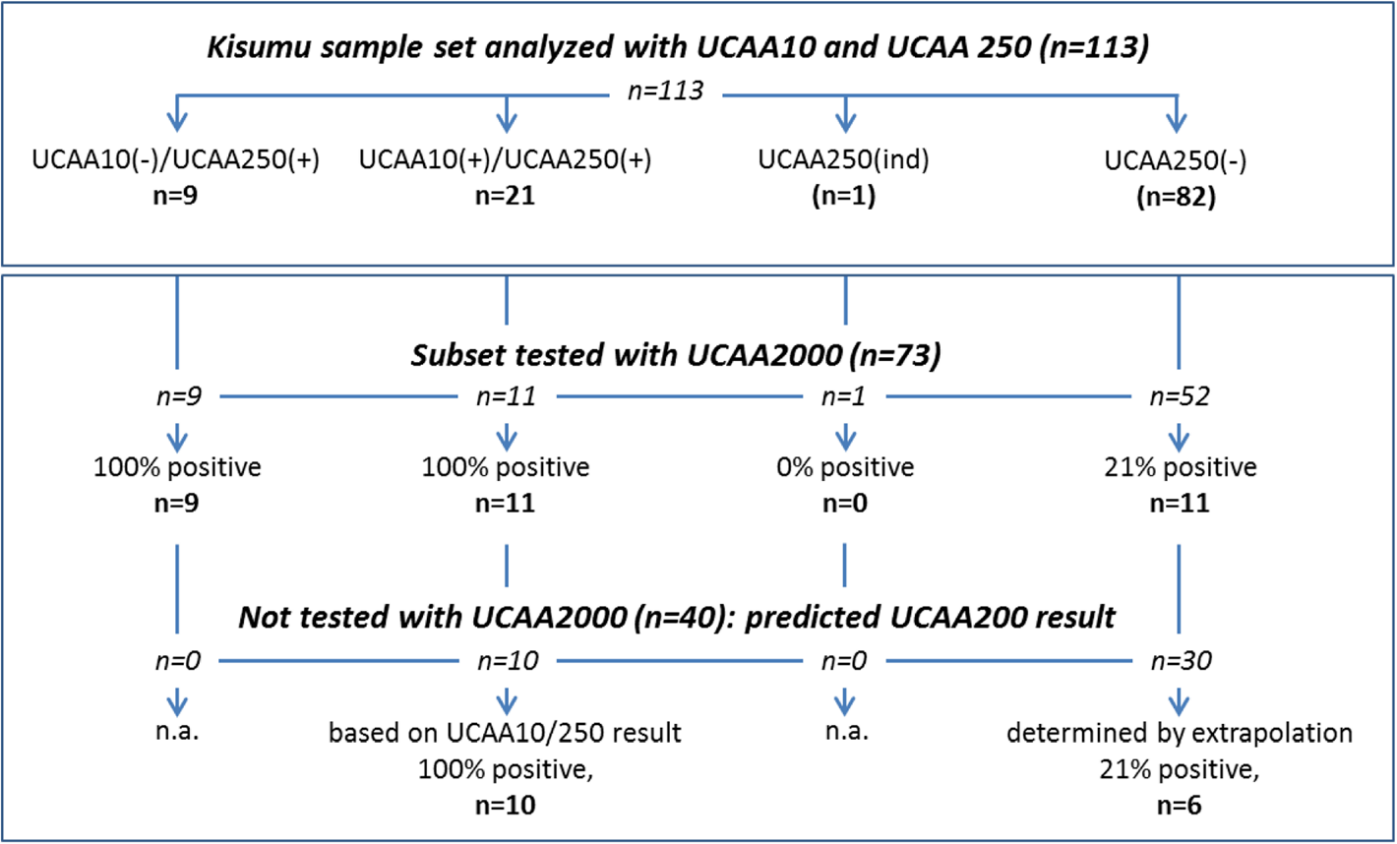
Expected qualitative UCAA2000 test results for samples tested with UCAA10 and UCAA250. The Kisumu sample set (n = 113) was tested with both the UCAA10 and UCAA250 assay. Only a non-random subset (n = 73; selection based on UCAA10/250 results) was tested with the UCAA2000 assay. The subset included 52 samples with a negative UCAA250 result. Ten samples with a positive test result for both UCAA10 and UCAA250, plus 30 samples with a negative test result for the UCAA250 were excluded. Extrapolation: for the 30 negatives, the same percentage of positives (21%) was assumed as empirically determined for the 52 negatives tested with the UCAA2000 assay. Numbers between brackets indicate samples with a negative test result; (+) and (−) following the UCAA assay indicate a positive or negative test result, respectively.

Note that the subset of 73 samples does not represent an absolute random selection of the full Kisumu set. The subset did not include 30 out of 82 samples that had tested negative with the UCAA10 and UCAA250 assay. Also not included were 10 out of 21 positive samples that demonstrated high reactivity with both the UCAA10 and −250 assay and undoubtedly would return a positive test result with the UCAA2000 (Figure 4).

### Kato Katz analysis of the ***S. mansoni*** stool set

Stool samples collected at three consecutive days were analysed in duplicate at KEMRI/CDC (Kisumu, Kenya). Egg counts were quantified on two slides of 42 mg per stool per day as described by Katz *el al.* [34], resulting in 6 KK counts per individual; Table 2 summarizes the results. Stool egg counts are shown to vary over three consecutive days, resulting in prevalences ranging from 11-15% per day, with 22% for the 3x2 KK results (25 egg-positive cases). The number of eggs counted per duplicate KK (84 mg stool) varied between 2 to 14 eggs, i.e. 24 to 168 epg, a universal finding for low endemic regions. Of the 25 egg positives, 16 were identified with the UCAA10 assay, and 18 with the UCAA250. The UCAA2000 assay was applied on a subset containing 17 of the 25 egg positives, and identified 16 out of these positive. Hence, using the predetermined UCAA2000 QC cutoff threshold (a CAA level of 0.3 pg/mL), 1 egg positive (2 eggs identified in only one of the three duplicate KK, translating to just 8 epg) was missed. Using the concentration based assay in our laboratory on well documented banked samples (unpublished results), no egg positives were missed when testing serum (500 μL serum, SCAA500 [26]). When testing larger urine sample collections with the UCAA2000 assay, the ‘failure rate’ never exceeded 5% including potential administrative error.

**Table 2:** **Kato Katz analysis, two slides per stool on 3 stools collected on consecutive days**

	***Day 1***	***Day 2***	***Day 3***	***Day 1-3***
***Assay result*** ^***a***^	***KK1-2***	***KK3-4***	***KK5-6***	***KK1-6*** ^***b***^
***KK egg positive***	17 (15%)	13 (12%)	12 (11%)	25 (22%)
***KK egg negative***	96 (85%)	100 (88%)	101 (89)	88 (78%)
***Samples tested***	113	113	113	113

^a^Number of individuals with/without eggs; between brackets the percentage of samples with a specific test result considering testing the complete collection (positive egg result indicates prevalence of infection by KK).

^b^Total number of individuals with eggs; identified an egg in at least one out of six slides.

### Definition of a gold standard and clinical specificity

Similar to the approach defining a composite reference standard as recommended by WHO/TDR [39], in this study true positives are defined as being positive in KK and/or the UCAA2000 assay (note: ten samples positive for both, UCAA10 and UCAA250, were not tested with larger sample volume but counted as UCAA2000 positive). Targeting 100% specificity, samples identified as UCAA2000 indecisive (potential CAA levels between 0.1 and 0.3 pg/ml) are interpreted as negatives. Calculation of the clinical sensitivity and prevalence of the full sample set (113 samples) required extrapolation to obtain a qualitative UCAA2000 test result for the 30 UCAA250 negatives which could not be tested with the UCAA2000 assay. The extrapolation was based on the UCAA2000 assay result obtained with 52 UCAA250 negatives (a random selection from all 82 UCAA250 negatives in the full set of 113 samples) of which 11 samples (21%) returned a positive test result in the UCAA2000, thus predicting 6 additional positives in the set of 30 (Figure 4). Moreover, 10 additional samples with both, a positive UCAA10 and UCAA250 result, but not tested with the UCAA2000 assay were also regarded as UCAA2000 positive (Figure 4). Table 3 shows the sensitivity and prevalence determined for the 113 samples that were tested with the KK and the UCP-LF CAA assays (UCAA10, UCAA250 and UCAA2000). According to the above defined gold standard the Kisumu set (n = 113) contained 48 true positives. This number includes 17 CAA positives identified with the UCAA2000 assay that were negative with the UCAA250 and UCAA10, and 1 egg positive identified only by detection of two eggs in one out of six slides. As indicated by the presence of samples generating a UCAA2000 indecisive result, it might be possible that with a further increase of the sample volume (for instance using the UCAA7500 assay) a few more positives could be identified. The 17 samples identified by UCAA2000 only, indicated CAA levels in the range of 0.3 to 3 pg/mL. It is yet unknown what level of CAA in urine can be expected for individuals infected by single worm pair, but we note that the currently maintained UCAA2000 lower detection level of 0.3 pg/mL CAA in urine is >10-fold lower in comparison to the 3–10 pg/mL CAA in serum predicted to indicate an infection by a single worm (see references in Corstjens *et al.* [26]). According to the model by de Vlas *et al.* [20], based on KK egg count the true prevalence would be in the 40-60% range; the UCAA2000 assay with a prevalence of 41.6% falls within that range. In addition, the use of larger concentration devices allows convenient confirmation of the positives through analysis of larger sample volumes. It is important to note that the various UCP-LF assays require only a single urine sample for analysis, whereas accurate identification by KK requires analysis of multiple stool samples, which puts heavy logistical demands on national mapping programmes and may reduce compliance considerably. Compared to egg positives identified by KK, the difference is obvious: 41.6% positives for the UCAA2000 assay requiring only a single urine sample, versus 22.1% positives for the 6-fold KK analysis on three consecutive-day stool samples. A single stool duplicate KK (2 slides) showed a prevalence as low as 9.7%, less than half of what was found by the UCAA10 (using 10 μL urine, not requiring a concentration device). The 26.5% prevalence determined with UCAA250 assay was already significantly higher than the prevalence with the 6-fold KK, both indicating the value and efficacy of the UCP-LF CAA assay.

**Table 3:** **Clinical sensitivity and prevalence for UCP-LF assays and KK analysis**

***Kisumu set: N = 113***	***True positives*** ^***a***^	***False negatives*** ^***b***^	***Sensitivity***	***Prevalence***
***Gold Standard***	48	0	100%	42%
***UCAA10***	21	27	44%	19%
***UCAA250***	30	18	63%	27%
***UCAA2000***	47^c^	1	98%	42%
***KK1-2***	17	31	35%	15%
***KK3-4***	13	35	27%	12%
***KK5-6***	11	37	23%	10%
***KK1-6***	25	23	52%	22%

^a^The Gold Standard positive is defined as a sample (stool or paired urine) with at least a single egg in the 6-fold KK stool analysis or a CAA level > 0.3 pg/mL in urine as determined with the UCAA2000 assay.

^b^False negatives are defined against the Gold Standard and calculated by ‘False Negatives = 48 – True Positives’.

^c^Number of UCAA2000 positives (including 16 additional positives, Figure 4).

In this study, the urine UCP-LF CAA assays showed to be highly superior in clinical sensitivity compared to KK. Using larger sample volumes increased the number of CAA positive samples from 21 for the UCAA10 assay (10 μL urine) to 30 for the UCAA250 assay (250 μL urine), to 47 for the UCAA2000 assay (2,000 μL urine). Before this test could claim ‘gold standard’ status, more investigation is required with a focus on samples generating a UCAA negative result with a positive KK result (in this study applying to only a single case). Administrative errors at any stage of the sampling and testing cannot be excluded, and therefore retesting requiring follow-up sample collection (possibly including serum) is necessary to unequivocally confirm discrepant results with KK and any other test.

## Conclusions

The UCP-LF CAA assay to identify active *Schistosoma* infections utilizing urine samples demonstrated excellent sensitivity. Analysis of a single 10 μL urine sample clearly demonstrated superiority to a duplicate KK performed on a single stool sample: 19% versus 10-15% prevalence respectively for UCP-LF (UCAA10) and KK. The implementation of a concentration step allowed 200-fold larger sample input (UCAA2000) which further improved CAA detection sensitivity from 30 pg/mL to 0.3 pg/mL reaching a prevalence of 42% while maintaining specificity at 100%. As the test is genus specific and has the highest demonstrated sensitivity showing infection levels similar to the true prevalence predicted by the statistical model of de Vlas *et al.* [20], the UCP-LF CAA concentration assay for urine is a very attractive alternative for egg count assays (stool and/or urine) especially in low endemic settings approaching interruption of transmission and elimination of schistosomiasis. Moreover, the non-invasive one-stop sample collection, omission of a cold-chain for assay reagents (shipping and storage) and expectedly also for the collected urine samples, are desirable features for acceptance and implementation of the assay in resource limited settings.

## Abbreviations

AWA: Adult worm antigen
CAA: Circulating anodic antigen
CCA: Circulating cathodic antigen
epg: Eggs per gram
LF: Lateral flow
NHU: Normal human urine
POC: Point-of-care
QC: Quality control
Sn: Sensitivity
TCA: Trichloroacetic acid
UCP: Upconverting phosphor
